# Perception of musculoskeletal pain in the state of confinement: associated factors

**DOI:** 10.1590/1518-8345.4894.3454

**Published:** 2021-06-28

**Authors:** Carlos Carpintero-Rubio, Bárbara Torres-Chica, María Alexandra Guadrón-Romero, Laura Visiers-Jiménez, David Peña-Otero

**Affiliations:** 1 Colegio Profesional de Practicantes de Reconstrucción Postural, Estrasburgo, France.; 2 Clínica Sarua, Madrid, Spain.; 3 Hospital de Sierrallana, Servicio Cántabro de Salud, Torrelavega, Cantabria, Spain.; 4 Universidad Cátolica de Ávila, Escuela Técnico Profesional en Ciencias de la Salud, Clínica Mompía, Cantabria, Spain.; 5 Cómité Ético de Investigación con Medicamentos de Cantabria, CEI-CEIm, Santander, Cantabria, Spain.; 6 Instituto de Investigación-Grupo de Enfermería-Sanitaria Gregorio Marañón, IiSGM, Madrid, Spain.; 7 Hospital de Sierrallana, Subdirección de Cuidados, Servicio Cántabro de Salud, Torrelavega, Cantabria, Spain.; 8 Instituto de Investigación-Grupo de Enfermería-Sanitaria Valdecilla, IDIVAL, Santander, Cantabria, Spain.

**Keywords:** Pain, Quarantine, Pandemics, Coronavirus Infections, Risk Factors, Home Health Nursing, Dor, Quarentena, Pandemias, Infecções por Coronavirus, Fatores de Risco, Enfermagem Domiciliar, Dolor, Cuarentena, Pandemias, Infecciones por Coronavirus, Factores de Riesgo, Cuidados de Enfermería en el Hogar

## Abstract

**Objective:**

to describe the perception of musculoskeletal pain in the population and how the state of confinement (adopted as a measure to control contagion by COVID-19) has interfered with it, as well as identifying the sociodemographic, occupational, physical, and psychosocial factors involved.

**Method:**

an observational, cross-sectional and analytical study, with simple random probabilistic sampling, aimed at residents in Spain over 18 years old during the confinement period. An *ad hoc* survey was conducted, consisting in 59 items.

**Results:**

a total of 3,247 surveys were answered. Persistent musculoskeletal pain or significant episodes thereof increased 22.2% during confinement. The main location was the spine (49.5%). The related factors were decreased physical activity, increased seated position, and use of electronic devices. The psychological impact of confinement was also related to the perception of musculoskeletal pain.

**Conclusion:**

the state of confinement causes an increase in the perception of musculoskeletal pain. The identification of a particularly sensitive population profile, as well as that of the related factors, allows establishing multidisciplinary approaches in health promotion.

## Abstract (pt)

**Objetivo:**

descrever a percepção da dor musculoesquelética na população e como o estado de confinamento (adotado como medida de controle do contágio pela COVID-19) tem interferido na mesma, bem como identificar os fatores sociodemográficos, ocupacionais, físicos e psicossociais envolvidos.

**Método:**

estudo observacional, transversal e analítico, com amostragem probabilística aleatória simples, realizado com residentes na Espanha, maiores de 18 anos, durante o período de confinamento, para tanto, foi realizada uma enquete *ad-hoc* com 59 itens.

**Resultados:**

foram recebidas 3.247 respostas. Dor musculoesquelética persistente ou episódios significativos da mesma aumentaram em 22,2% durante o confinamento. A localização principal foi a coluna vertebral (49,5%). Os fatores relacionados foram a diminuição da atividade física, o aumento da posição sentada e o uso de dispositivos eletrônicos. O impacto psicológico do confinamento também esteve relacionado à percepção de dor musculoesquelética.

**Conclusão:**

o estado de confinamento acarreta aumento na percepção da dor musculoesquelética. A identificação de um perfil populacional particularmente sensível, bem como dos fatores relacionados, permite estabelecer abordagens multidisciplinares na promoção da saúde.

## Abstract (es)

**Objetivo:**

describir la percepción de dolor musculoesquelético en la población y cómo el estado de confinamiento (adoptado como medida de control de contagio por COVID-19) ha interferido en la misma, así como identificar los factores sociodemográficos, laborales, físicos y psicosociales implicados.

**Método:**

estudio observacional, transversal y analítico, con muestreo probabilístico aleatorio simple, dirigido a residentes en España, mayores de 18 años durante el periodo de confinamiento. Se realizó una encuesta *ad-hoc* compuesta por 59 ítems.

**Resultados:**

se cumplimentaron 3247 encuestas. El dolor musculoesquelético persistente o los episodios significativos del mismo se incrementaron un 22,2% durante el confinamiento. La principal localización fue el raquis (49,5%). Los factores relacionados fueron la disminución de la actividad física, el aumento de la posición sentada y del uso de dispositivos electrónicos. El impacto psicológico del confinamiento también se relacionó con la percepción de dolor musculoesquelético.

**Conclusión:**

el estado de confinamiento provoca un incremento en la percepción de dolor musculoesquelético. La identificación de un perfil poblacional especialmente sensible, así como la identificación de los factores relacionados, permite establecer abordajes multidisciplinares en la promoción de la salud.

## Introduction

Musculoskeletal pain has a high prevalence in the population and some of its manifestations such as low back pain or neck pain are among the main causes of disability worldwide^(^ 1 ^-^ 3 ^)^. Its prevention and treatment constitute an important social and health challenge due to the deterioration that it generates in quality of life, the labor costs that it causes, and the health care required by people who suffer from it^(^ 4 ^-^ 5 ^)^.

Pain is an unpleasant sensory and emotional experience, associated with actual or potential tissue damage. It is subjected to the subjectivity of those who suffer it^(^ 6 ^)^ and is multi-factorial, which requires a biopsychosocial and interdisciplinary approach^(^ 7 ^)^. In musculoskeletal pain there are multiple elements that can be involved, from damage in tissues of the locomotor system that triggers nociceptive pain, to others of a neuropathic or psychosocial nature. The latter influence the perception and experience of pain. Chronification of the painful experience can lead to central sensitization and allodynia^(^ 8 ^)^.

To minimize transmission of the SARS-CoV-2 coronavirus, contain the progression of the COVID-19 disease and strengthen the public health system, on March 14th, 2020, the Spanish Government declared the State of Alarm throughout the Spanish territory, according to Royal Decree 463/2020^(^ 9 ^)^. Among the containment measures adopted was limiting the movement of people through public spaces, a situation that was strictly maintained until the entry into force of the “Plano de Desescalada” [De-escalation Plan] approved on April 28th, 2020. In such a prolonged state of confinement, several elements can favor the appearance of musculoskeletal pain episodes or increase them if they are already present.

On the one hand, physical inactivity, which causes atrophy of the skeletal muscles and supporting connective tissues^(^ 10 ^-^ 11 ^)^. A pathognomonic relationship has been suggested between the severity of muscle atrophy and the development, for example, of low back pain^(^ 11 ^)^. Apart from that, sedentarism and immobility are factors that increase the stiffness of tendons, fasciae, ligaments, and muscles. Muscle stiffness has also been related to pain in conditions such as low back pain and neck pain^(^ 12 ^-^ 13 ^)^. Another negative effect associated with sedentarism has to do with the impairment of somatosensory stimulation in the locomotor system. Poor proprioceptive stimulation favors the development of dystonias^(^ 14 ^-^ 15 ^)^ and of changes in neuromuscular control, situations that can cause excess muscle tension, restrictions in joint mobility, overloads and, pain^(^ 16 ^)^. In addition, as a consequence of sedentarism, body weight tends to increase, something that also conditions the perception of pain. It is known that the symptomatic treatment of overweight people lasts longer than that of normal weight subjects^(^ 17 ^)^, in addition to requiring higher analgesic doses^(^ 6 ^)^. During confinement there have been changes in the eating habits and behaviors mainly characterized by the increase in the intake of hypercaloric products^(^ 18 ^)^, which promotes an increase in the Body Mass Index (BMI)^(^ 19 ^)^.

Another trigger for musculoskeletal pain is poor posture habits. Remote work or a leisure model based on the consumption of multimedia content and the use of mobile devices, favor the maintenance of deficient ergonomic positions during sustained periods, which can cause overloads and pain^(^ 20 ^)^.

On the other hand, the implementation of extemporaneous exercises or sports activities, a generalized situation during confinement as an alternative to the usual physical activity^(^ 21 ^)^, can constitute another situation that generates overloads, injuries, and pain. The recommendations and advice focused on maintaining physical fitness have been very numerous during this period, so that a large number of people have begun to perform activities without proper conditioning or exceeding their functional abilities^(^ 22 ^)^.

Finally, factors of a psychological nature such as anxiety or catastrophism also negatively modify the perception of pain^(^ 23 ^-^ 25 ^)^. Confinement has made it necessary to combine family care, remote work and domestic tasks, an unusual and complex reality for many families that has been able to increase the levels of anxiety and stress in both the child and adult populations. To this situation, on the one hand, a high degree of economic and labor uncertainty has been added^(^ 26 ^-^ 27 ^)^ and, on the other, the fear and insecurity generated by living with a health crisis of planetary magnitude, whose epidemiological data at a global level are alarming. The fear of resuming social interaction and habits prior to the pandemic can also increase stress, anxiety, and depression in the population^(^ 28 ^)^.

In view of all the above, the objective of this study was to describe the perception of musculoskeletal pain in the population and how the state of confinement (adopted as a control measure for contagion by COVID-19) has interfered with it, as well as to identify the sociodemographic, labor, physical and psychosocial factors involved.

## Method

An observational, cross-sectional and analytical study, with simple random and probabilistic sample, conducted in Spain. The recruitment period and field study with dissemination and subsequent answer to the survey was from May 1^st^ to May 11^th^, 2020.

The population studied consisted in individuals over 18 years old living in Spain. The selection criteria adopted were the following: people over 18 years of age living in Spain, with access to an electronic device with Internet (computer, tablet, mobile phone, etc.) and who voluntarily agreed to participate in the study after being invited to collaborate by answering a questionnaire (from May 1^st^ to May 11^th^, 2020) sent through public and private institutions to the general population, after approximately 2 months of home confinement established throughout the Spanish territory (started on March 14^th^, 2020).

They were grouped into three blocks: sociodemographic data, pain, and related factors (physical and psychological) before and during the confinement period; the sociodemographic variables were as follows: age, gender, weight, height, marital status, nationality, autonomous community of residence, schooling level, employment status, income level, place of work, outside space of the home, and number of people in the household; pain and related factors (before and/or during the confinement period): perception of the status of the musculoskeletal system, suffering from ailment of the musculoskeletal system, pain duration, pain location, pain intensity (current, weekly average, worst pain), interference of pain with other activities, coping strategies for pain, attending Physiotherapy and Nursing appointment, daily hours of use of electronic devices, daily hours in a seated position, time of sports activity, type of sports activity, frequency of activity sports, feeling of effort during sports activity, perception of restlessness or impatience, perception of fatigue, perception of concentration, perception of irritability/fatigue, perception of sleep disorder, and concern about these symptoms.

The measurement instrument used to carry out the study was an anonymous on-line questionnaire consisting in 59 *ad hoc*-prepared questions, through Google Forms platform. It was designed in its entirety by the researchers, due to the specificity of the situation to be studied, although it was previously piloted to guarantee both the understanding of the questions and the answers included and the mean duration required for its completion. The final questionnaire was distributed through social networks (mainly WhatsApp, Twitter, Facebook, and Instagram) and the International Nursing Network (INN), and was sent by email to the Spanish Professional Associations of Nursing and Physiotherapy. It was also published by the Cantabrian Health Service in the SCSalud APP. In addition, a press release was published in the web of the *Enfermería en Desarrollo* journal, encouraging its readers to fill out the survey and forward it to their contacts.

Data collection was carried out based on the study variables from the answers indicated in the completed surveys received.

Calculation of the sample size was based on the total Spanish population over 18 years of age (39,047,503 individuals), registered as of January 1^st^, 2020 at the Spanish National Institute of Statistics, considering a Type I error <5% and a confidence level of 95%. A minimum of 2,401 participants was required.

Data was analyzed using the IBM SPSS v.22 program. The continuous variables were described using measures of central tendency (mean) and measures of dispersion (standard deviation); while the categorical variables were described through absolute and relative frequency tables. Before and during confinement, the baseline characteristics shown by the study participants according to variables of severity and complications were compared. The comparison of categorical variables was carried out using the Chi-Square test, and that of continuous variables, by means of the Student’s t test. The 95% confidence intervals were determined using the standard methods.

The study was approved by the Cantabrian CEI-CEIm (Code 2020.195). At all times, the Standards of Good Clinical Practice and the current legislation regarding biomedical research (Law 14/2007 on Biomedical Research, of July 3^rd^) were observed. The treatment, communication, and transfer of personal data of all the participants was in accordance with the provisions of the applicable regulations (Regulation (EU) 2016/679 of the European Parliament and of the Council, of April 27^th^, 2016, General Regulation of Data Protection (*Reglamento General de Protección de Datos, RGPD*) and Organic Law 3/2018, of December 5th, for the Protection of Personal Data and guarantee of the digital rights).

## Results

A total of 3,247 surveys were received. The sociodemographic characteristics of the participants are presented in Table 1.

**Table 1:** Frequency estimates for the sociodemographic variables (n=3,247). Spain, 2020

VARIABLES	CATEGORIES	n* (%^†^)
Gender	Female	2,324 (71.6)
	Male	923 (28.4)
Marital status	Married/Domestic partner	1,785 (55.0)
	Separated/Divorced	271 (8.3)
	Single	1,058 (32.6)
	Widower	45 (1.4)
	Other	88 (2.7)
Nationality	Spanish	3,179 (97.9)
	Other	68 (2.1)
Schooling level	PhD	85 (2.6)
	Post-graduate training	484 (14.9)
	University studies	1,346 (41.5)
	Vocational training/Bachelor's degree	1,056 (32.5)
	Basic studies (EGB^‡^, ESO^§^)	270 (8.3)
	No studies	6 (0.2)
Employment situation prior to confinement	Exclusive dedication to home and/or family care	137 (4.2)
	Unemployed	236 (7.3)
	Employed by others	2,128 (65.5)
	Self-employed	258 (7.9)
	Retiree	270 (8.3)
	Other	218 (6.7)
Change in the employment situation during confinement	None	1,354 (41.7)
	Remote work	889 (27.4)
	Workday reduction	89 (2.7)
	ERTE^||^	419 (12.9)
	Other	496 (15.3)
Annual gross salary	No income	313 (9.6)
	Less than €12,000	511 (15.7)
	Between €12,001 and €20,000	853 (26.3)
	Between €20,001 and €30,000	803 (24.7)
	Between €30,001 and €50,000	590 (18.2)
	Between €50,001 and €100,000	163 (5.0)
	More than €100,000	14 (0.4)
Perception of economic income of the family unit	Unburdened	1,406 (43.3)
	Tight	1,398 (43.1)
	With difficulties to make ends meet	287 (8.8)
	I need to ask for some kind of help	70 (2.2)
	Serious problems to make ends meet	86 (2.6)
Workplace before confinement	From my home	70 (2.2)
	Outside my home	2,547 (78.4)
	I do not work	630 (19.4)
Housing (garden, outdoor terrace, etc.)	Yes	2,109 (65.0)
	No	1,138 (35.0)
Number of people in the household during confinement (including you)	1	546 (16.8)
	2	965 (29.7)
	3	799 (24.6)
	4	735 (22.6)
	More than 4	(6.2)

* n = Sample size; % = Statistical frequency;

‡ EGB = Enseñanza General Básica (Basic General Education);

§ ESO = Educación Secundaria Obligatoria (Compulsory Secondary Education);

|| ERTE = Expediente de Regulación Temporal de Empleo (File of Temporary Employment Regulation)

Regarding the musculoskeletal system, only 48.5% of the surveyed participants considered that their health status prior to confinement was good. 47.2% asserted suffering constant pain or significant episodes before this period, with 57.7% of them lasting more than 6 months and the most common locations being the spine (51.2%) and the lower limbs. 63.5% of the participants perceived that the confinement situation worsened their musculoskeletal health status, attributing it to a reduction in regular physical or sports activity in 80.6% of the cases. During the confinement period, the percentage of participants who reported having constant pain or significant episodes thereof increased by 22.2%. However, the percentage values of its most common locations remained similar: spine (49.5%) and lower limbs (Table 2).

**Table 2:** Frequency estimates of the main locations of perceived pain before and during the confinement situation (n=3247). Spain, 2020

MAIN LOCATIONS OF PERCEIVED PAIN		BEFORE n* (%^†^)	DURING n* (%^†^)
TOTAL OF PARTICIPANTS WITH PAIN		n*= 1,534 (47.2)	n*= 2,253 (69.4)
Head		71 (4.63)	180 (7.99)
Spine	Cervical	299 (19.51)	422 (18.73)
	Dorsal (central part of the back)	133 (8.68)	216 (9.59)
	Lumbar	353 (23.03)	478 (21.21)
Lower limb (hip, leg...)		296 (19.31)	410 (18.19)
Upper limb (shoulder, arm...)		220 (14.35)	249 (11.05)
Chest/Abdomen		10 (0.65)	37 (1.64)
Other locations		152 (9.92)	261 (11.58)

* n = Sample size;

† % = Statistical frequency

35.1% of those surveyed reported a pain intensity between 5 and 7 Visual Analogue Scale (VAS)^(^ 29 ^)^ points (moderate-intense pain) when answering the questionnaire, with a similar average pain in 36.6% and a maximum intensity of 7-9 points (intense-very intense) in 38.9% of the participants.

During the confinement situation, the time of use of electronic devices increased, as well as the time that the respondents remained seated, while the time of physical activity was reduced, increasing only in the anaerobic modality, which rose from 8.9% to 13.1%. Despite everything, the performance of physical exercises with aerobic characteristics continued to predominate (30.4%). Sports activity began to be carried out more constantly throughout the week, but with less duration and intensity. Table 3 presents the data related to the physical factors studied before and during confinement.

**Table 3:** Frequency estimates of the associated physical factors before and during the confinement situation (n=3,247). Spain, 2020

VARIABLES	CATEGORIES	BEFORE	DURING
		n* (%^†^)	n* (%^†^)
Daily time spent on electronic devices (television, computer, tablet, mobile, videogames...) for leisure and/or work (n*=3,247)	1 hour	247 (7.6)	55 (1.7)
	Between 1 and 2 hours	912 (28.1)	264 (8.1)
	Between 2 and 5 hours	1,124 (34.6)	1,034 (31.8)
	Between 5 and 8 hours	425 (13.1)	881 (27.1)
	Between 8 and 10 hours	355 (10.9)	565 (17.4)
	More than 10 hours	156 (4.8)	436 (13.4)
	None	15 (0.5)	8 (0.2)
	Other	13 (0.4)	4 (0.1)
Daily time in a seated position (n*=3,247)	1 hour	155 (4.8)	25 (0.8)
	Between 1 and 2 hours	675 (20.8)	173 (5.3)
	Between 2 and 5 hours	1,118 (34.4)	852 (26.2)
	Between 5 and 8 hours	685 (21.1)	901 (27.7)
	Between 8 and 10 hours	485 (14.9)	690 (21.3)
	More than 10 hours	101 (3.1)	589 (18.1)
	None	15 (0.5)	11 (0.3)
	Other	13 (0.4)	6 (0.2)
Type of sports activity (n*=3,247)	Aerobic (walking, running, swimming, riding a bicycle...)	1,821 (56.1)	988 (30.4)
	Anaerobic (weight lifting, crossfit...)	288 (8.9)	424 (13.1)
	Yoga or Pilates	402 (12.4)	471 (14.5)
	None	581 (17.9)	1,115 (34.3)
	Other	155 (4.8)	249 (7.7)
Days/week of sports activity		n*= 2,666	n*= 2132
	1 day	202 (7.58)	123 (5.77)
	2 days	566 (21.23)	238 (11.16)
	3 days	725 (27.20)	420 (19.70)
	4 days	454 (17.03)	310 (14.54)
	5 days	414 (15.53)	396 (18.57)
	6 days	131 (4.91)	289 (13.56)
	7 days	174 (6.53)	356 (16.70)
Daily time of sports activity		n*= 2,666	n*= 2,132
	Less than 1 hour	737 (27.64)	1,233 (57.83)
	Between 1 and 2 hours	1,691 (63.43)	821 (38.51)
	More than 2 hours	197 (7.39)	58 (2.72)
	Other	41 (1.54)	20 (0.94)
Perception of training intensity		n*= 2,666	n*= 2,132
	Soft	820 (30.76)	943 (44.23)
	Moderate	1,267 (47.52)	880 (41.28)
	A little hard	412 (15.45)	221 (10.37)
	Hard	124 (4.65)	68 (3.19)
	Very hard	22 (0.83)	10 (0.47)
	Other	21 (0.79)	10 (0.47)

* n = Sample size;

† % = Statistical frequency

The participants who reported pain before confinement considered that it did not interfere with any of their activities (37.1%) and, if it did, it mainly limited their sports (36.9%) or work (25.9%) activities or carrying out household chores (25.3%). The main strategies used to cope with pain were drug treatment (analgesics, muscle relaxants, etc.) in practically all of the respondents (97.6%), attendance to a specialized consultation (45.7%) or stretching (44.0%) or doing some sports activity (35.4%), either in isolation or in combination, while a minority sought advice for pain management on the Internet or in self-help books (1.5%). Pain during confinement mainly interfered with the performance of household chores (38.9%) and of sports activities (28.4%), using stretching (54.0%) and use of medications (50.6%) as coping strategies, either in isolation or in combination, while only a minority sought advice for pain management on the Internet or in self-help books (3.5%) or consulted a specialist (4.4%).

On the other hand, it should be noted that 35.6% of the participants stated that, before confinement, they regularly experienced restlessness or impatience; 33.0%, muscular tension; 29.7%, fatigability or tiredness; and 28.2%, sleep disorders. 32.3% of the respondents did not report having any symptoms on a regular basis. In addition, of the 67.7% who regularly felt any symptoms before confinement, 28.8% were not concerned at all if the symptoms would disappear, compared to 43.0% who were a little concerned and 25.3% who were moderately concerned. In this sense, the percentage of participants who stated suffering from regular psychosocial symptoms during confinement, in addition to pain, increased significantly (p<0.05), reaching a percentage of 88.0% of the total respondents. In turn, the number of people who perceived symptomatic worsening increased in all the symptoms analyzed (Table 4), consequently increasing the concern about whether these symptoms would disappear.

**Table 4:** Frequency estimates of the perception of psychosocial symptoms derived from the confinement situation (n=3,247). Spain, 2020

	PSYCHOSOCIAL SYMPTOMS n* (%^†^)					
	RESTLENESS IMPATIENCE	FATIGABILITY TIREDNESS	CONCENTRATION	IRRITABILITY	MUSCLE TENSION	SLEEP HABITS
**It has worsened**	1,731 (53.3)	1,523 (46.9)	1,526 (47.0)	1,612 (49.6)	1,675 (51.6)	1,894 (58.3)
**It has improved**	337 (10.4)	484 (14.9)	260 (8.0)	328 (10.1)	335 (10.3)	269 (8.3)
**It has remained unchanged**	1,179 (36.6)	1,240 (38.2)	1,461 (45.0)	1,307 (40.3)	1,237 (38.1)	1,084 (33.4)

* n = Sample size;

† % = Statistical frequency

In relation to the physiotherapy consultation, before confinement 14.5% of the respondents attended regularly and 32.1% did so punctually. During confinement, only 14.8% of these attended with the usual frequency and 65.2% did not attend any appointment. 3.6% of the participants attended the Nursing consultation regularly before confinement and 10.9%, punctually. Of these, 57.9% attended the Nursing consultation during confinement with the usual frequency.

According to the data presented in Table 5, the relationship between the variables studied and pain before and during confinement is statistically significant (p<0.05). In turn, the existence of a positive correlation between pain during confinement and all the sociodemographic, physical, and psychosocial factors studied is observed (Table 5).

**Table 5:** Frequency estimates, correlations and statistical significance between pain and sociodemographic, physical, and psychosocial factors according to the subjects grouped in factors before and during confinement (n=3,247). Spain, 2020

FACTOR		PAIN BEFORE		p^‡^	PAIN DURING		p^‡^	r^§^
		YES n* (%^†^)	NO n* (%^†^)		YES n* (%^†^)	NO n* (%^†^)		
Sociodemographic. cultural and work-related								
Gender	Female	1.151 (35.4)	1.173 (36.1)	**0.07**	1.485 (45.8)	839 (25.7)	**0.01**	0.114
	Male	383 (11.8)	540 (16.6)		475 (14.6)	448 (13.9)		
BMI^||^	Normal (18.5-25)	721 (22.2)	995 (30.6)	**0.01**	983 (30.3)	733 (22.6)	**0.01**	0.069
	Overweight (>25-30)	545 (16.8)	520 (16)		667 (20.5)	397 (12.2)		
	Obesity (>30)	267 (8.2)	199 (6.1)		308 (9.5)	159 (4.9)		
Marital status	With a partner	877 (27)	908 (28)	**0.02**	1.097 (33.8)	688 (21.2)	**0.01**	0.025
	No partner	659 (20.3)	803 (24.7)		862 (26.5)	600 (18.5)		
Age	18 to ≤65 years old	1.455 (44.8)	1.634 (50.3)	**0.05**	1.874 (57.7)	1.215 (37.4)	**0.05**	0.030
	>65 years old	78 (2.4)	80 (2.4)		85 (2.6)	73 (2.2)		
Schooling level	With higher education	835 (25.7)	1.080 (33.3)	**0.01**	1.102 (34)	813 (25)	**0.01**	0.068
	Without higher education	697 (21.4)	635 (19.6)		865 (26.6)	467 (14.4)		
Wage	Up to 30.000	1.211 (37.3)	1.269 (39.1)	**0.01**	1.543 (47.5)	937 (28.8)	**0.01**	0.088
	More than 30.000	321 (9.8)	446 (13.7)		415 (12.8)	352 (10.9)		
Perception of economic income	Without difficulties	1.279 (39.4)	1.519 (46.8)	**0.01**	1.670 (51.4)	1.134 (34.9)	**0.01**	0.089
	With difficulties	249 (7.7)	200 (6.1)		290 (8.9)	153 (4.7)		
Housing (with garden. terrace. etc..)	Yes	997 (30.7)	1.112 (34.2)	0.8	1.231 (37.9)	878 (27)	**0.01**	0.054
	No	536 (16.5)	602 (18.5)		728 (22.4)	410 (12.6)		
People in the household	≤3	1.106 (34.1)	1.204 (37.1)	**0.01**	1.405 (43.3)	905 (27.9)	**0.03**	0.017
	>3	426 (13.1)	511 (15.7)		553 (17)	384 (11.8)		
**Physical**								
Daily use time of electronic devices	Less than 8 hours	1391 (42.8)	1345 (41.4)	**0.01**	1311 (40.4)	935 (28.8)	**0.01**	0.059
	More than 8 hours	233 (7.2)	278 (8.6)		647 (19.9)	354 (10.9)		
Daily time in a seated position	Less than 8 hours	1260 (38.8)	1401 (43.2)	**0.01**	1137 (35)	831 (25.6)	**0.01**	0.065
	More than 8 hours	273 (8.4)	313 (9.6)		822 (25.3)	457 (14.1)		
Type of sports activity (n before=2.666; n during=2.132)	Aerobic	877 (32.9)	1024 (38.4)	**0.01**	618 (29)	495 (23.2)	**0.01**	0.078
	Anaerobic	143 (5.4)	220 (8.2)		251 (11.8)	297 (13.9)		
	Yoga and/or Pilates	245 (9.2)	157 (5.9)		323 (15.2)	148 (6.9)		
Daily time of sports activity (n before=2.666; n during=2.132)	Less than 1 hour	392 (14.7)	386 (14.5)	**0.01**	754 (35.4)	499 (23.4)	**0.01**	0.082
	More than 1 hour	866 (32.5)	1022 (38.3)		458 (21.5)	421 (19.7)		
Perception of training intensity (n before=2.666; n during=2.132)	Soft to moderate	1066 (40)	1043 (39.1)	**0.01**	1107 (51.9)	726 (34)	**0.01**	0.180
	Hard to extreme	198 (7.4)	360 (13.5)		104 (4.9)	195 (9.1)		
Coping strategies for pain (n before=1534; n during=2253))	Pharmacological	793 (51.7)		**0.01**	1195 (53.0)		**0.01**	0.149
	Non-pharmacological	741 (48.3)			1058 (47.0)			
**Psicosociales**								
Síntomas psicosociales	Yes	1274 (39.2)	936 (28.8)	**0.01**	1858 (57.2)	1003 (30.9)	**0.01**	0.259
	Did not feel any symptom	258 (8)	779 (24)		100 (3.1)	286 (8.8)		

* n = Sample size;

† % = Statistical frequency;

‡ p = Level of statistical significance;

§ r = Pearson's correlation obtained for the result between factors before and after confinement;

|| BMI = Body Mass Index

## Discussion

The respondents were mainly women (71.6%), with a mean age of 43.75 years old [Standard Deviation (SD)=12.71], of Spanish nationality (97.9%) and with a mean BMI of 25.91 (SD=10.64), in the lower limit of overweight. In general, the participants had a partner and university studies, and were active at work prior to confinement, carrying out their professional activity outside their homes. To facilitate discussion, data prior to confinement and those corresponding to that period will be independently analyzed.

*Musculoskeletal pain prior to confinement and associated factors*. The results obtained in relation to the main location of musculoskeletal pain converge with the epidemiological data published to date, which place low back pain and neck pain among the ten disorders with the highest incidence in the world population^(^ 30 ^)^. The least symptomatic locations were chest, abdomen, and head. It should be noted that, in more than half of the cases, pain was chronic, that is, lasting more than 6 months^(^ 31 ^)^, and with high intensity. These data reveal a problem that is often underestimated^(^ 32 ^-^ 33 ^)^. The low percentage of individuals who, despite living with severe symptoms, resorted to Physiotherapy or Nursing professionals to receive treatment or consult their ailments is noteworthy. Chronic pain is especially striking in the aged population, where incidence is very high^(^ 34 ^-^ 35 ^)^. The data obtained in this paper coincide with this reality, reflecting a greater impact of musculoskeletal pain in the advanced age groups (over 65 years old).

Among the sociodemographic indicators that show a relationship with the musculoskeletal pain perceived before confinement, the following stand out: gender, with women experiencing more habitual pain; age and BMI, which are directly proportional to perceived pain; and level of studies and salary. Having high academic training, as well as high income, make it possible, on the one hand, to manage information related to health in an efficient manner, as well as to make use of unsubsidized health coverage. Both elements can justify minimizing the impact of musculoskeletal pain in this segment of the population. On the other hand, unskilled jobs carry a higher level of workload and physical demand than skilled jobs, which could have a negative effect on the musculoskeletal level. However, some characteristics of highly qualified jobs such as sedentarism or stress could be considered equally harmful to the locomotor system^(^ 36 ^)^.

Regarding the relationship of pain with physical activity, the results obtained indicate that maintaining a regular level of activity constitutes an effective strategy in pain management^(^ 37 ^)^. In addition, high intensity training was more effective in pain control than light training. Individuals capable of high intensity training may have allostatic adaptations that increase their resistance to physical stress^(^ 38 ^)^, although too intense a training load could cause cumulative harms to the musculoskeletal system^(^ 39 ^)^.

In the analyzed population, the presence of numerous psychosocial factors favoring the development of musculoskeletal symptoms was found, namely: restlessness, impatience, irritability, lack of concentration, fatigability, and sleep disorders. These elements are clearly related to usual pain in the population studied. The contextual factors of a psychosocial nature are valued in the management of musculoskeletal pain, coming to be considered as “yellow flags” on which social health care should fall^(^ 8 ^)^. In certain conditions such as chronic nonspecific low back pain, cognitive-behavioral treatment has come to be proposed as a priority therapeutic line^(^ 40 ^)^.

*Evolution of pain during confinement and associated factors*. In general terms, the incidence of musculoskeletal pain increased during the confinement period, with the main affected body regions remaining unchanged. From a sociodemographic point of view, the participants who lived as a couple were the most affected, especially women. In many cases, the effort to reconcile professional obligations and domestic tasks has been added to the continuous care of children, dependent family members, support in schoolwork, as well as the need to share physical spaces and electronic resources with the family members. From a gender perspective, this situation has fallen mainly on women, and the existing gap has been reinforced^(^ 41 ^)^. It is likely that this situation, rather than having a direct impact on the physical load, has triggered or increased stressors of a psychological nature clearly related to the perception of pain.

With regard to physical and sports activity, the musculoskeletal pain perceived during confinement shows a clear association with the increase, first of all, in the use of electronic devices (more than 8 hours a day); secondly, by staying in a seated position (more than 8 hours a day); and, finally, meager sports practice (less than 1 hour a day). These elements can be considered indicators of sedentarism^(^ 42 ^)^, a condition that causes, among other disorders, muscle atrophy and of the supportive skeletal tissues, increased myofascial stiffness, somatosensory deficits and, linked to all of the above, musculoskeletal pain^(^ 43 ^)^. During confinement, the practice of anaerobic activities and disciplines such as Yoga or Pilates increased, while the practice of aerobic activities decreased.

It is worth noting the benefit of having a garden or terrace at home in relation to the perception of musculoskeletal pain. A space with these characteristics invites people to maintain an adequate regimen of physical activity by offering more possibilities than closed and reduced spaces, which has positive repercussions on pain and quality of life, without forgetting other psychological or emotional benefits.

In general, the strategies used to combat musculoskeletal pain during confinement have consisted of pharmacological treatments. This can be due to difficulties in traveling outside the home to receive other types of treatments as a consequence of mobility restrictions, and this is demonstrated by the reduction in the number of Physiotherapy or Nursing appointments during this period. The most used non-pharmacological strategy was muscle stretching followed by the application of cold or heat. This indicates, on the one hand, the increased perception of muscle tension in the participants, something that could be related to psychological factors such as stress or sleep disorders, as well as to an increase in sedentary behaviors and prolonged posture maintenance^(^ 44 ^-^ 45 ^)^. On the other hand, the perception of inflammation, hence the therapeutic resource of cryotherapy. These non-traumatic inflammatory processes can derive from the adoption of certain postures for a long time, for example, the seated position^(^ 46 ^)^. However, a traumatic origin of these conditions cannot be ruled out since, on numerous occasions, sports activities began to be practiced within the home inspired by generic recommendations from social networks or television programs^(^ 47 ^)^. It is possible that people were not sufficiently conditioned for this type of exercise or that the basic recommendations for a good practice without risk of injury were not followed.

The onset, in some cases, and the increase in others, of the psychological symptoms in the population studied during confinement is very striking, that is, the influence that both the pandemic and the associated confinement have had on the emotional and behavioral stability of people^(^ 18 ^)^. A number of research studies during previous infectious outbreaks have revealed psychological repercussions on the population^(^ 48 ^)^. Feelings of loss of control and of being trapped in confinement are likely to substantially intensify the symptoms^(^ 48 ^)^. It is also necessary to highlight, as unavoidable, instability and uncertainty at the work level (a large majority of the participants were forced to work remotely, suffered some contractual regulation, or were fired), as well as the need to combine work/school obligations and recreational activities for all members of the family nucleus at home. In many cases, the insufficiency or obsolescence of computer equipment and Internet coverage would have to be added to the aforementioned, something that would only increase the levels of tension and perceived stress.

Among the study limitations are both sample dispersion and female predominance. However, the high number of answers obtained makes it possible to define numerous features of the Spanish population that usually perceive musculoskeletal pain, as well as the influence that confinement has had on it.

The present study provides new evidence on the high prevalence of musculoskeletal pain in the healthy population, as well as its complex multi-factoriality. It has been proven that many of the causal factors involved in the onset or aggravation of this type of symptoms are inherently present in a state of home confinement such as that which occurred during the COVID-19 pandemic in 2020.

The results obtained in this study will make it possible to adapt health promotion and prevention strategies from a biopsychosocial perspective that ultimately improve the quality of life of the population. Likewise, these could be extrapolated internationally, across populations with similar characteristics, given that the pandemic continues to require more or less restrictive confinement measures worldwide, in order to contain the spread of the virus.

## Conclusion

Confinement has caused an increase in the perception of lumbar and cervical pain in women, especially in those over 65 years of age, with the following related factors: reduction in the intensity and duration of aerobic physical activity, increase in the use of electronic devices, increase in the permanence in a seated position, and worsening of the psychosocial symptoms.

The definition of a population profile that is especially sensitive to the impact of confinement with regard to the perception of musculoskeletal pain, as well as the identification of the causal factors involved in such perception, will allow establishing multidisciplinary approaches in health promotion.
